# Refractory adult-onset Still’s disease complicated with monoclonal gammopathy of undetermined significance: A case report

**DOI:** 10.1097/MD.0000000000032107

**Published:** 2022-12-09

**Authors:** Kenji Saito, Tomoyuki Asano, Haruki Matsumoto, Yuya Fujita, Naoki Matsuoka, Hiroshi Ohkawara, Yuya Sumichika, Shuhei Yoshida, Jumpei Temmoku, Makiko Yashiro-Furuya, Shuzo Sato, Hiroshi Watanabe, Kiyoshi Migita

**Affiliations:** a Department of Rheumatology, Fukushima Medical University School of Medicine, Fukushima, Japan; b Department of Hematology, Fukushima Medical University School of Medicine, Fukushima, Japan.

**Keywords:** adult-onset Still’s disease, interleukin-18, interleukin-6, monoclonal gammopathy of undetermined significance, multiple myeloma

## Abstract

Adult-onset Still’s disease (AOSD) is a rare inflammatory disease characterized by a classic triad of daily spike fever, arthritis, and a typical salmon-pink rash. The involvement of inflammatory cytokines by various factors such as infection, drug, or neoplasm causes refractory AOSD.

**Patient concerns:**

We report a 63-year-old man with a high fever, rash, hyperferritinemia, and M proteinemia. His serum levels of interleukin-6 and interleukin-18 were remarkably high at 192 and 114,250 pg/mL, respectively.

**Diagnosis:**

AOSD complicated with monoclonal gammopathy of undetermined significance was diagnosed.

**Interventions:**

After steroid pulse therapy followed by oral prednisolone, cyclosporin, methotrexate, and colchicine, serum ferritin levels temporarily declined, but secondary cytomegalovirus infections exacerbated AOSD’s activity.

**Outcomes:**

Finally, after tocilizumab induction, AOSD activity was gradually suppressed over a long period.

**Lessons:**

The disease activity of AOSD is exacerbated by multiple factors, including comorbidities or infections. Clinicians need to consider that monoclonal gammopathy of undetermined significance complications might become AOSD refractory by an elevation of the inflammatory cytokines. Moreover, further prospective studies are required to confirm this result.

## 1. Introduction

Adult-onset Still’s disease (AOSD) is a rare systemic inflammatory disorder of unknown origin, characterized by high spiking fever, rash, and arthritis.^[1]^ Because of the increase with serum proinflammatory cytokines^[2,3]^ and the clinical effectiveness of anti-cytokine therapy,^[4]^ the overproduction of various proinflammatory cytokines represents the pathological characteristics of the disease. Especially if we consider that the serum levels of interleukin (IL)-6^[3]^ and IL-18^[5]^ correlate with AOSD activity, its high levels made the patient’s condition refractory.^[6]^ Meanwhile, monoclonal gammopathy of undetermined significance (MGUS) is a preneoplastic statement characterized by the presence of product serum M-protein < 3.0 g/dL, bone marrow clonal plasma cells < 10%.^[7]^ IL-6 levels are significantly elevated in the serum of patients with MGUS compared with healthy controls,^[8]^ and IL-6 plays an essential role in the proliferation of human B lymphocytes and myeloma cells.^[9]^ Many cases associated with inflammatory rheumatic disease and neoplasms have been reported. However, no reports of AOSD with MGUS have been reported to date.

## 2. Case report

**Figure 1.**
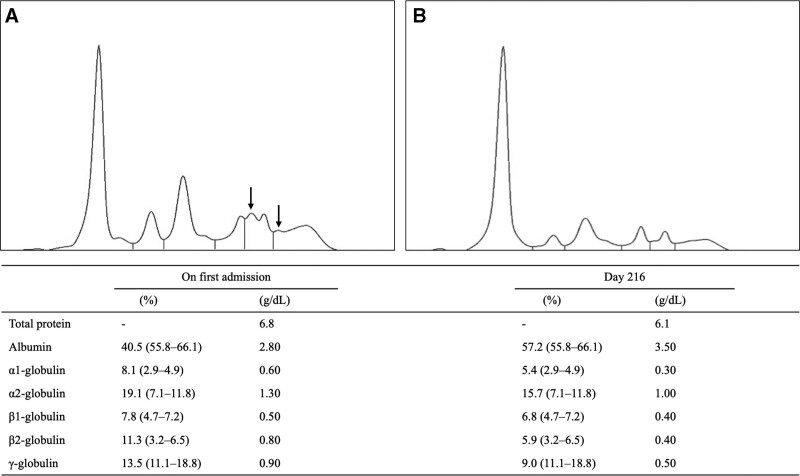
Findings of protein electrophoresis before and after the treatment. (A) An abnormal protein was seen in the β1, β2, and γ regions before the treatment (arrow). (B) Abnormal proteins disappeared after the treatment on day 60.

A 63-year-old Japanese male was referred to and first hospitalized in our department due to a fever of unknown origin and systemic erythema in October 2020. Two weeks before he came to our hospital, he visited a local clinic because of a 5-days-lasting remittent fever, myalgia, night sweating, and erythema on the trunk. He had no notable family history. Both influenza virus and coronavirus-19 antigen tests were negative. He was given an antipyretic analgesic, but it did not relieve his fever. Seven days after his symptom onset, fexofenadine, tranexamic acid, and corticosteroid ointment were administered to him, but his high fever and erythema were not improved. Finally, he was referred to our department 14 days after the onset of his symptoms. His height was 156 cm, and his weight was 54 kg. His body temperature was high at 38.9°C; blood pressure (123/80 mmHg) and pulse rate (79/min) were normal. Physical examination showed muscle pain in his biceps and biceps femoris. Irregular erythema with unclear boundaries was found on his abdomen, waist, and upper and lower extremities. Laboratory data are shown in Table 1, which demonstrated increased white blood cells, neutrophils, C-reactive protein, and ferritin. Mild liver dysfunction was also observed. His renal function was normal. Serum IL-6 and IL-18 were remarkably high at 192 pg/mL and 114,250 pg/mL, respectively. Immunoglobulin A (IgA) showed a slight increase in gamma globulin’s light chain. Protein electrophoresis revealed that abnormal protein existed in the β1, β2, and γ regions (Fig. 1A). Although the serum free light chain ratio was in the normal range, the κ and λ light chains were increased to 23.2 mg/L (normal range, 3.30–19.40), 82.6 mg/L (normal range, 5.70–26.30), respectively. Additional immune-electrophoresis showed serum M proteinemia (IgA-λ type) and urinary Bence–Jones protein at the λ-region.

**Table 1:** Laboratory findings on admission.

Variable	Value	Normal range	Variable	Value	Normal range
Peripheral blood			Blood urea nitrogen	16 mg/dL	(8–22)
White blood cells	15,500/µL	(2800–8800)	Creatinine	0.70 mg/dL	(0.4–0.7)
Neutrophils	90%	(44–74)	β2 microglobulin	2.06 mg/dL	(<2.00)
Lymphocytes	5%	(20–50)	Sodium	139 mg/dL	(138–145)
Monocytes	4%	(1–14)	Chloride	101 mg/dL	(3.6–4.8)
Eosinophils	1%	(0–6)	Potassium	8.7 mg/dL	(8.8–10.1)
Basophils	0%	(0–1)	Fe	29 µg/dL	(43–172)
Red blood cells	4.30 × 10^6^/µL	(3.66–4.78)	UIBC	217 µg/dL	(137–325)
Hemoglobin	13.6 g/dL	(11.6–14.0)	Ferritin	15,024 ng/mL	(12–60)
Hematocrit	40.4%	(34.1–41.7)	C-reactive protein	8.21 mg/dL	(<0.30)
MCV	94.0 fL	(81.8–97.2)	ESR (1 h)	40 mm	(3–15)
Platelets	29.5 × 10^4^/µL	(14.7–34.1)	IgG	849 mg/dL	(870–1700)
Blood chemistry			IgG4	17 mg/dL	(5–117)
Total protein	6.6 g/dL	(6.7–8.3)	IgA	792 mg/dL	(110–410)
Albumin	2.8 g/dL	(3.9–4.9)	IgM	79 mg/dL	(35–220)
α1-globulin	8.1%	(2.9–4.9)	κ-chain	23.2 mg/L	(3.30-19.40)
α2-globulin	19.1%	(7.1–11.8)	λ-chain	82.6 mg/L	(5.70-26.30)
β1-globulin	7.5%	(4.7–7.2)	κ/λ ratio	0.28 mg/L	(<1.650)
β2-globulin	11.3%	(3.2–6.5)	ANA	1:160 (Gr)	(<1:159)
γ-globulin	13.5%	(11.8–18.8)	SAA	924.9 µg/mL	(<8.0)
Total bilirubin	0.3 mg/dL	(0.2–1.2)	Soluble IL-2 receptor	936 U/mL	(121–613)
Aspartate transaminase	57 U/L	(13–33)	Infection		
Alanine transaminase	114 U/L	(6–27)	HBs Ag	Negative	
Lactate dehydrogenase	442 U/L	(119–229)	HCV Ab	Negative	
Alkaline phosphatase	308 U/L	(115–359)	β-D glucan	<6.0 pg/mL	(<11.0)
γ-Glutamyltranspeptidase	41 U/L	(10–47)	IGRA	Negative	

ANA = anti-nuclear antibodies, ESR = erythrocyte sedimentation rate, HBs = Ag hepatitis B virus surface antigen, HCV Ab = anti-hepatitis C virus antibody, Ig = immunoglobulin, IGRA = interferon-gamma release assay for Mycobacterium tuberculosis, MCV = mean corpuscular volume, SAA = serum amyloid A, UIBC = unsaturated iron-binding capacity.

He underwent a bone marrow biopsy because we suspected that he might have had some hematological disorder. Histological findings of the specimen showed that gamma-positive cells represent < 10%, and the light chain represents a bias (λ:κ = 4:1). Bone marrow cells were differentiated correctly in all 3 lines, and we identified a collection of plasma cell-like cells. Immunostaining for these plasma cells was positive for CD56 and Cyclin D1. Finally, he had a diagnosis of AOSD based on Yamaguchi’s criteria,^[10]^ complicated with light chain MGUS.^[11]^ The patient’s condition had not progressed to multiple myeloma (MM) at the diagnosis and final follow-up. Furthermore, other autoinflammatory/autoimmune diseases were excluded.

**Figure 2.**
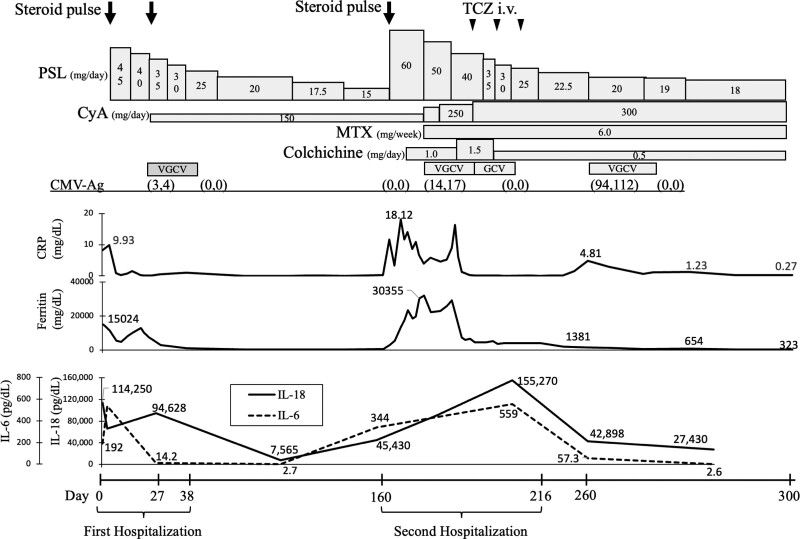
The clinical course of the present case. CMV-Ag = cytomegalovirus antigenemia, CRP = C-reactive protein, CyA = cyclosporine, GCV = ganciclovir, IL = interleukin, i.v. = intravenous injection, MTX = methotrexate, PSL = prednisolone, TCZ = tocilizumab, VGCV = valganciclovir.

The clinical course is shown in Figure 2. Initially, he was treated with intravenous methylprednisolone (mPSL) for 3 days (1.0 g/day), followed by oral PSL at a 45 mg/day dose. Seven days after steroid therapy, serum ferritin levels were decreased to 5326 ng/mL, but AOSD remained highly active due to sustained hyperferritinemia. Furthermore, AOSD relapsed after cytomegalovirus (CMV) infection (using human CMV pp65 Antigen ELISA test) on day 24, and serum ferritin levels rose to 12,823 ng/mL. Secondary intravenous mPSL (1.0 g/day) with valganciclovir for CMV infection was administered to him. In addition to mPSL, cyclosporine (CyA) (150 mg/day) was added on day 27. After immunosuppressive therapies, ferritin levels steadily decreased to 190 ng/mL. Levels of IL-6 and IL-18 were also reduced. So he was discharged on day 38 and was followed up for 4 months without relapse of AOSD.

On April 2021 (on day 160), because of AOSD relapsed with high fever, arthritis, erythema, liver dysfunction, hyperferritinemia (30,355 mg/dL), and elevated CRP (18.12 mg/dL), he was secondarily hospitalized in our hospital. As a result, he was treated with third steroid pulse therapy (1.0 g/day), followed by oral CyA (300 mg/day) and oral methotrexate (6.0 mg/week). Because colchicine has been reported efficient for refractory AOSD,^[12]^ we added colchicine at 1.5 mg/day. However, the patient developed diarrhea: a side effect of colchicine; therefore colchicine dose was reduced to 0.5 mg/day. Same as his first hospitalization due to AOSD, hyperferritinemia was prolonged with CMV infection, and intravenous tocilizumab (8 mg/kg/2 weeks) improved his symptoms, such as high fever, arthritis, and erythema. After 3 courses of tocilizumab injection, it was discontinued due to hypogammaglobulinemia. Finally, the abnormal protein shown in protein electrophoresis on the first visit disappeared (on day 216) (Fig. 1B). In addition, the M-protein seen in immune-electrophoresis had disappeared. Two years after the first onset, no relapse of AOSD or no malignant conversion from MGUS to MM was observed.

## 3. Discussion

Herein, we present a case of AOSD complicated with MGUS. The patient had persistent hyperferritinemia after treatment with glucocorticoids and immunosuppressants. Because MGUS is generally a benign monoclonal gammopathy, the complication with AOSD may seem like a coincidence. However, because the elevation of proinflammatory cytokines, such as IL-6 and IL-18 in MGUS patients, has been reported previously,^[13]^ we should consider the etiological interaction between MGUS and AOSD.

AOSD is a systemic autoinflammatory disease characterized by spiking high fever, rash, and arthritis, that develops in which innate and acquired immunity interact to induce a cytokine storm.^[14]^ Although their pathogenesis remains unclear, macrophage activation syndrome (MAS) might be triggered by immunological stimuli such as bacteria, viruses, chemicals, or neoplasms; moreover, the human leukocyte antigen or familial Mediterranean fever gene polymorphisms might modify these triggers.^[15–17]^ These danger signals are transmitted through Toll-like receptors on macrophages’ surfaces to intense production of IL-18 and IL-1β.^[18]^ In addition, it is known that many inflammatory cytokines, including IL-18, are elevated in the serum of AOSD patients during the active phase of the disease with MAS.^[19,20]^ In particular, IL-18 plays a key inflammatory cytokine that acts upstream of IL-6, tumor necrosis factor-α, or interferon -γ in the cytokine cascade.^[21]^ In addition, serum IL-18 exhibits abnormally high levels associated with symptoms of AOSD, such as fever, arthritis, and erythema.^[22]^

Meanwhile, MM is a disease characterized by the monoclonal proliferation of plasma cells and increased products, such as monoclonal immunoglobulin (M-protein), in serum or urine.^[23]^ In principle, MGUS (premalignancy conditions of symptomatic MM) and smoldering (asymptomatic) MM should be followed up without treatment. Systemic chemotherapy is considered only when these conditions shift to symptomatic MM. Furthermore, patients with MGUS develop the disease condition at 12% after 10 years, and 30% after 25 years, respectively.^[24]^ Although our patient showed an increase in serum free light chain, the bone marrow findings showed that the risk of myeloma was very low, and he was followed up without chemotherapy against malignant neoplasm.

IL-6 is one of the central inflammatory cytokines in the pathogenesis of MM because it promotes neoplastic plasma cell proliferation and inhibits apoptosis.^[25]^ Therefore, IL-6 signal suppression via an anti-IL-6 receptor antibody is essential in controlling plasma cell tumors. Expression levels of the IL-6Rα chain (CD126) were higher than normal controls in patients with MGUS and MM.^[26]^ However, clinical trials of siltuximab, an anti-IL-6 monoclonal antibody against MM, did not improve the long-term prognosis of patients, and it showed that the effect of the anti-IL-6 antibody agent was limited.^[27]^ Conversely, IL-18, an IL-1 cytokine family, is involved in tumor induction by inducing interferon-gamma.^[28,29]^ In particular, high levels of IL-18 in the bone marrow in patients with MM were suggested to be independent determinants of reduced overall survival, thus supporting the hypothesis that IL-18 contributes to the progression of MM.^[30]^

As shown in the previous paragraph, AOSD and MM are pathologically involved in IL-6 and IL-18, and anti-cytokine therapy trials for both diseases have been done. Unfortunately, the effect of the anti-IL-6 antibody on MM was only limited, and its therapeutic efficacy was not proven.^[27]^ However, tocilizumab, an anti-IL-6R antibody against refractory AOSD, has been proven to have a high inhibitory effect on disease activity and an excellent persistence rate.^[31]^ Furthermore, inhibitors against IL-1β, such as anakinra and canakinumab, which belong to the same family of IL-18, are also effective against AOSD and juvenile idiopathic arthritis.^[32,33]^ Moreover, new agents against AOSD have suggested clinical efficacy during the clinical trials with rilonacept; the IL-1 inhibitor,^[34]^ or taking alfa; the IL-18 binding proteins.^[35]^

It had been reported that MGUS was occasionally associated with rheumatic diseases, such as systemic lupus erythematosus, Sjögren’s syndrome, and rheumatoid arthritis.^[36]^ Moreover, a small number of cases with AOSD and malignancy have also been reported.^[37]^ Hofheinz et al found 47 articles about complications of AOSD and malignancy; 50% had hematopoietic disorders, and 50% had solid tumors.^[37]^ According to their report, 33% of AOSD symptoms disappeared after treatment of malignant tumors, and the risk factor of malignant tumors in AOSD was elderly onset, atypical rash, high lactate dehydrogenase titer, atypical cells in the different blood counts, and high levels of sIL-2R.^[37]^ This is the first case report of AOSD and MGUS complications to our knowledge. Because MGUS is the most common hematopoietic premalignant condition, it is essential to consider the common mechanisms and pathogenesis between rheumatic disease and MGUS.

However, we have one more point to discuss in this case report. We have encountered twice attacks of AOSD with MAS. These twice attacks of MAS were considered to be due to the onset (or relapse) of AOSD activation, but CMV infection occurred concurrently after each immunosuppressive therapy. In particular, serum IL-6 and IL-18 levels were markedly elevated after the last CMV infection. CMV infection occurs in patients with primary immunodeficiency disease or after intensive immunosuppressive therapy.^[38]^ In addition, CMV infection often triggers MAS and is therefore considered one of the causes of AOSD.^[39]^ Anti-cytomegalovirus IgM antibody or CMV-DNA titer has been reported to be significantly higher in the serum of AOSD patients, suggesting that CMV infection is involved in triggering the onset and relapse of AOSD.^[40]^ One of the limitations of this case report is that it is unclear whether the elevation of IL-6 and IL-18 was due to active AOSD or CMV infection. However, serum inflammatory cytokine measurements in AOSD patients with CMV infection showed an increase in tumor necrosis factor -α but not IL-6 and IL-18.^[40]^

In conclusion, the complication of MGUS may become AOSD intractable. Clinicians should be aware of the inflammatory cytokine-releasing status that may be enhanced by coexisting hematologic disorders such as MGUS.

## Acknowledgments

We thank Enago (http://enago.jp) for the English language review.

## Author contributions

**Conceptualization:** Kenji Saito, Tomoyuki Asano, Shuzo Sato.

**Data curation:** Tomoyuki Asano.

**Formal analysis:** Tomoyuki Asano.

**Funding acquisition:** Tomoyuki Asano.

**Investigation:** Kenji Saito, Tomoyuki Asano, Haruki Matsumoto, Yuya Fujita, Naoki Matsuoka, Hiroshi Ohkawara, Yuya Sumichika, Shuhei Yoshida.

**Methodology:** Kenji Saito, Jumpei Temmoku, Makiko Yashiro-Furuya, Shuzo Sato.

**Project administration:** Tomoyuki Asano.

**Resources:** Tomoyuki Asano, Haruki Matsumoto, Yuya Fujita, Naoki Matsuoka, Hiroshi Ohkawara.

**Supervision:** Tomoyuki Asano, Shuzo Sato, Hiroshi Watanabe, Kiyoshi Migita.

**Validation:** Hiroshi Watanabe, Kiyoshi Migita.

**Visualization:** Tomoyuki Asano, Shuzo Sato, Kiyoshi Migita.

**Writing – original draft:** Kenji Saito.

**Writing – review & editing:** Tomoyuki Asano.
